# An unusual diarrheal outbreak in the community in Eastern Thailand caused by Norovirus GII.3[P25]

**DOI:** 10.1186/s12985-024-02296-z

**Published:** 2024-01-19

**Authors:** Patpong Udompat, Krongkan Srimuang, Pawinee Doungngern, Nattakarn Thippamom, Sininat Petcharat, Khwankamon Rattanatumhi, Sirorat Khiewbanyang, Pantila Taweewigyakarn, Somkid Kripattanapong, Sasiprapa Ninwattana, Ananporn Supataragul, Spencer L. Sterling, Chonticha Klungthong, Khajohn Joonlasak, Wudtichai Manasatienkij, Thomas S. Cotrone, Stefan Fernandez, Supaporn Wacharapluesadee, Opass Putcharoen

**Affiliations:** 1 Phrapokklao Hospital, Chanthaburi, 22000 Thailand; 2 Thai Red Cross Emerging Infectious Diseases Clinical Center, King Chulalongkorn Memorial Hospital, Rama IV Road, Pathumwan, Bangkok, 10330 Thailand; 3 Division of Epidemiology, Department of Disease Control, Ministry of Public Health, Muang, Nonthaburi, 11000 Thailand; 4 Faculty of Medicine, Chulalongkorn University, Bangkok, 10330 Thailand; 5 Henry M. Jackson Foundation, Bethesda, MD USA; 6 Department of Virology, Armed Forces Research Institute of Medical Sciences, Bangkok, 10400 Thailand; 7 Division of Infectious Diseases, Department of Medicine, Faculty of Medicine, Chulalongkorn University, Bangkok, 10330 Thailand

**Keywords:** Acute gastroenteritis, Norovirus, Outbreak, Dual-typing, Next Generation Sequencing

## Abstract

**Background:**

Sentinel laboratory surveillance for diarrheal disease determined norovirus to be the most common cause of non-bacterial gastroenteritis in people during the COVID-19 pandemic in Thailand. An increase in patients presenting with diarrhea and vomiting in hospitals across Chanthaburi province between December 2021 and January 2022 led to the need for the identification of viral pathogens that may be responsible for the outbreak.

**Methods:**

Fecal samples (rectal swabs or stool) from 93 patients, of which 65 patients were collected during the December 2021 to January 2022 outbreak, were collected and screened for viral infection by real-time RT-PCR. Positive samples for norovirus GII were then genotyped by targeted amplification and sequencing of partial polymerase and capsid genes. Full genome sequencing was performed from the predominant strain, GII.3[P25].

**Results:**

Norovirus was the most common virus detected in human fecal samples in this study. 39 of 65 outbreak samples (60%) and 3 of 28 (10%) non-outbreak samples were positive for norovirus genogroup II. One was positive for rotavirus, and one indicated co-infection with rotavirus and norovirus genogroups I and II. Nucleotide sequences of VP1 and RdRp gene were successfully obtained from 28 of 39 positive norovirus GII and used for dual-typing; 25/28 (89.3%) were GII.3, and 24/28 (85.7) were GII.P25, respectively. Norovirus GII.3[P25] was the predominant strain responsible for this outbreak. The full genome sequence of norovirus GII.3[P25] from our study is the first reported in Thailand and has 98.62% and 98.57% similarity to norovirus found in China in 2021 and the USA in 2022, respectively. We further demonstrate the presence of multiple co-circulating norovirus genotypes, including GII.21[P21], GII.17[P17], GII.3[P12] and GII.4[P31] in our study.

**Conclusions:**

An unusual diarrhea outbreak was found in December 2021 in eastern Thailand. Norovirus strain GII.3[P25] was the cause of the outbreak and was first detected in Thailand. The positive rate during GII.3[P25] outbreak was six times higher than sporadic cases (GII.4), and, atypically, adults were the primary infected population rather than children.

## Background

Sentinel laboratory surveillance for diarrheal illness has shown that norovirus has been one of the most common causes of non-bacterial gastroenteritis in Thailand and worldwide [1]. Noroviruses are non-enveloped, single-stranded positive-sense RNA viruses in the family *Caliciviridae*. The genome length ranges from 7.5 to 7.7 kb which contains three open reading frames (ORF); ORF1 encodes six nonstructural proteins (NS1/2, NS3, NS4, NS5, NS6, NS7). NS7 is an RNA-dependent RNA polymerase (RdRp) which is a key enzyme for viral replication [2]. ORF2 encodes the major structural protein VP1 which is divided into shell (S) and protruding (P) domains and is responsible for cell receptor interaction [3]. ORF3 encodes the minor structural protein VP2 which could serve as a VP1 helper protein and stabilization of the viral capsid.

Norovirus is globally endemic, with symptoms including stomach cramps, diarrhea, and vomiting that may last up to four days [4]. Severe norovirus disease typically occurs in infants, the elderly, and the immunocompromised and may require hospitalization. Norovirus is commonly transmitted via the fecal–oral route and can be foodborne, waterborne, airborne, person-to-person, or environmentally transmitted [5]. Disease susceptibility is dependent on multiple factors, including host genetics, infectious dose, and recency of past infections [4]. Generally, there are multiple strains of norovirus circulating at a single time, and the most common isolate from cases is considered the dominant strain. Dominant strains go through multi-year cycles of strain replacement, where mutations and recombination occur due to selective pressure from increased levels of immunity within the population [6].

The main mechanism of norovirus evolution is the recombination of genome junction regions which occurs between the RdRp region in ORF1 and the VP1 region in ORF2 and serves as the main factor of norovirus classification [7]. Based on 305 complete VP1 amino acids, the virus can be classified into 10 genogroups (GI–GX) which can be further subdivided into 49 genotypes. Recent discoveries have led to the tentative identification of two additional genogroups (GNA1 and GNA2) and three additional genotypes (GII.NA1, GII.NA2, and GIV.NA1). Norovirus genogroups GI, GII, GIV, GVIII, and GIX are known to infect and cause disease in humans. Further genome characterization based on RdRp nucleotide sequences can be classified into 8 P-groups and 2 tentative P-groups which can be further subdivided into 60 P-types and 14 tentative *P*-types [7]. Norovirus has extremely high rates of genetic diversity relative to other RNA viruses and increased rates of adaptation in variants. Norovirus genetic diversity is caused by the lack of 3’ exonuclease activity in the replication error-editing region of RNA polymerase, genetic recombination, and rapid selective pressures driven by the immune response of infected individuals. Previous studies on norovirus mutation rate within the VP1 gene from 64 unique molecular clones have estimated a mutation rate of 1.5 × 10^−4^ per nucleotide per cell infection [8].

In Thailand between 2000 and 2016, genogroup GII.4 was the most prevalent norovirus genotype (63.4%) in symptomatic individuals, followed by GII.3 (15.0%), GII.6 (3.9%), GII.17 (3.3%), and GII.13 (2.1%) [9]. From January 2015 to February 2017, norovirus investigations at two hospitals in Bangkok (n = 1468) and Khon Kaen province (n = 123) found genotype prevalence of GII.4 at 32.3% (64/198) and GII.17 at 11.6% (23/198), respectively. The recombination of norovirus also appeared sporadically, with GII.3[P12] at 8.6% (17/198) and GII.2[P16] at 40.4% (80/198) [10]. From 2017 to early 2019, GII.4 was the most frequently detected genotype (51.4%) in Bhumibol Adulyadej Hospital, Bangkok [11]. Norovirus GII.3[P25] accounted for one-third of outbreak cases in Chanthaburi Province, Thailand, from December 2021 to January 2022 [12].

During the COVID-19 pandemic, in which non-pharmaceutical interventions (NPIs) were widely used to prevent the spread of SARS-CoV-2, countries saw a significant decrease in communicable disease transmission, including noroviruses [13, 14]. Recent modeling efforts suggest that the drop in norovirus cases because of NPIs resulted in an increase of naïve individuals susceptible to norovirus [4]. Further, the relaxing of NPIs enforcement will likely lead to the slackening of hygiene measures that also reduce the risk of norovirus infection. The combination of increased susceptibility and decrease in preventive barriers suggests that there will be an increase in incidence of norovirus, although the scale to which that occurs is difficult to predict.

Data from Thailand’s event-based surveillance during 2017–2021 (internal data by the Department of Diseases Control, Ministry of Public Health, Thailand) determined norovirus outbreaks primarily occur between November and February, and 77% of outbreaks were found in schools, whereas other settings included prisons and among travelers. The most frequent outbreaks were among the 6–12 year age group, followed by 13–18 years and > 18 years. An outbreak of norovirus occurred in many hospitals across Chanthaburi province between December 2021 and January 2022, in which patients hospitalized with acute gastroenteritis and presented with diarrhea and vomiting or abdominal pain were submitted for viral characterization, epidemiology, and clinical analysis. This study extended the data from the previous study with different sources of specimens [12]. The present study aimed to analyze the genetic diversity of norovirus circulating in the Chanthaburi province during the December 2021–January 2022 outbreak and to assess the phylogenetic and phylodynamic features of norovirus GII strains identified during and after the outbreak.

## Material and methods

### Patient sample collection

A total of 93 stool samples and rectal swabs were collected between November 2021–September 2022 Among the samples collected during the study, 65 were collected during and independent outbreak from 26 December 2021 to 21 January 2022. Of these, 14 patients were healthcare workers, including 10 of 36 medical students and 4 of 12 healthcare workers who participated in the educational workshop at Phrapokklao Hospital from 23 to 25 December 2021. A retrospective cohort study of the healthcare workers was conducted to identify suspected sources. The cases were interviewed by phone. The demographic data, clinical symptoms, treatment outcome, food meals three days prior to symptom onset and related persons who had gastrointestinal symptoms were identified. Otherwise, we reviewed the medical records.

**Fig. 1:**
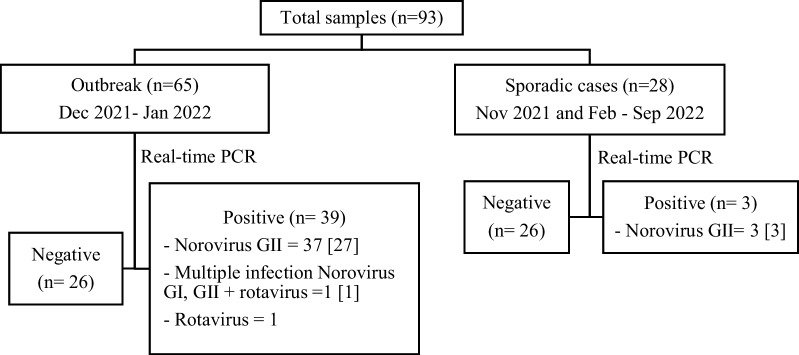
A diagram of the sample number of diarrhea virus detection results by real-time PCR. The number of samples that were successful in typing by PCR and Sanger sequencing was indicated in the square brackets [ ]

An additional 28 samples were collected from sporadic cases not linked to an outbreak cluster under the sentinel surveillance program of the Division of Epidemiology (DOE), Ministry of Public Health, including one case in November 2021 and 27 cases a following the December–January cluster of cases (Fig. 1). All samples were systematically collected following a standardized protocol by the DOE, specifically from patients with diarrhea at least 3 times within a 24-h period or watery diarrhea, approximately 3–5 samples per week per participating hospital.

Of the 93 patients in this study, 46 were male, and 47 were female. The median age of patients was 10 years (21 days–73 years). Age groups were categorized as follows: 21 days–10 years (n = 50), 11 years–20 years (n = 6), 21 years–30 years (n = 20), 31 years–40 years (n = 6), 41 years–50 years (n = 3), and above 50 years (n = 8) (Table 1). The most common symptoms were diarrhea (89.1%), nausea (67.2%) and abdominal pain (67.2%), but 4 patients had bloody diarrhea (4.3%). Eleven patients (11.8%) were admitted to a hospital. Mostly (79%) of the patients were the only one patient in their household and 43% of the patients reported history of visiting at least one of four large markets in Muang district. No specific food/shop was repeatedly mentioned by the patients.

**Table 1:** The demographic data

No. of participants	93	
Age, median (years)	10	
IQR	2–23	
Range	21 days–73 years	
*Age group*	*n (%)*	Positive^#^ (n = 42) [%]
< 10 years	50 (53.8)	21 [42.0%]
11–20 years	6 (6.5)	2 [33.3%]
21–30 years	20 (21.5)	9 [45.0%]
31–40 years	6 (6.5)	4 [66.7%]
41–50 years	3 (3.2)	2 [66.7%]
> 50 years	8 (8.6)	4 [50.0%]
*Sex*		
Male	46 (49.5)	20 [43.5%]
Female	47 (50.5)	22 [46.8%]

^#^Also include one case positive for norovirus GI, GII, and rotavirus and one case positive for rotavirus

All samples were collected by the Surveillance and Response Team (SRRT), Ministry of Public Health, in response to the widespread outbreak of diarrhea in Chanthaburi province from one provincial hospital and three suburban hospitals. Specimens were collected in a closed container within 24 to 72 h of symptom onset and kept refrigerated at 4 °C before being transported to the Emerging Infectious Diseases Clinical Center (EIDCC) at King Chulalongkorn Memorial Hospital for genetic testing. The samples used in this study are part of the outbreak investigation diagnosis; the Institutional Review Board (IRB) is not required.

### Nucleic acid extraction

The nucleic acid extraction was prepared by mixing 3.5 ml of a 10% (w/v) stool suspension with phosphate-buffered saline (1xPBS) with 1.5 ml glass beads in a 15 ml polypropylene tube and homogenized using a FastPrep-24 (MP Biomedicals, USA) instrument. After removing the solid particles by centrifugation at 4000×*g* for 5 min, supernatants were collected. Rectal swabs in viral transport medium (VTM) were mixed by vortexing for 2 min to ensure release of virions and genetic material and supernatant was collected. Total nucleic acids were extracted from a 400 µL supernatant sample using a magLEAD 12gC instrument (Precision System Science, Chiba, Japan) with a magLEAD Consumable Kit (Precision System Science) according to the manufacturer’s instructions to an elution volume of 50 µl.

### Real-time RT-PCR for the detection of six diarrhea viruses

Samples were initially tested by the Seegene Allplex GI-Virus Assay (Seegene Inc., Seoul, Republic of Korea) to identify cause of infection. This assay is a multiplex one-step real-time reverse transcription-polymerase chain reaction (RT-PCR) for simultaneously detecting rotavirus, norovirus GI and GII, adenovirus type 40/41, astrovirus, and sapovirus. Briefly, each 25 μl reaction containing 5 μl of nucleic acid was mixed with 20 μl of master mix, and real-time RT-PCR was performed using a CFX96 system (Bio-Rad, Hercules, CA, USA) under the following conditions: reverse transcription at 50 °C for 20 min, denaturation at 95 °C for 15 min, and 45 cycles of PCR (95 °C for 10 s, 60 °C for 1 min, and 72 °C for 30 s) for a total turnaround time of 2.5 h. The Seegene Viewer Software (Seegene Inc.) was used for data analysis. A result was considered positive when the PCR Cycle-threshold (Ct) curve was < 40, negative when the Ct was > 45, and indeterminate when the Ct was between 40 and 45.

### Target amplification and direct sequencing

Norovirus-positive samples were further characterized by target sequencing and phylogenetic analysis to determine genotype. cDNA was synthesized with random hexamer using SuperScript III reverse transcription kit (Invitrogen, Thermo Fisher Scientific, Massachusetts, USA) following the manufacturer’s instruction. The PCR reactions were performed by using the oligonucleotide primer, forward primer: MON431-F (5′-TGG ACI AGR GGI CCY AAY CA-3′), and reverse primer G2SKR-R (5′-CCR CCN GCA TRH CCR TTR TAC AT -3′), with the amplicon covering the partial RNA polymerase gene region and capsid region of size 570 bp [15]. The PCR protocol was performed as described. Briefly, PCR components included 0.4 µM of primer, 0.2 mM dNTP mix, 1.5 mM MgCl_2_, 10X PCR buffer, and 0.1 µl Platinum *Taq* DNA polymerase, and 2.5 μl of cDNA in a total volume of 25 µl. PCR was performed under the following conditions: initial denaturation at 94 °C for 5 min, followed by 40 cycles of 94 °C for 1 min, 50 °C for 1 min, and 72 °C for 1 min, with a final extension at 72 °C for 10 min, then hold at 4 °C indefinitely (modified from Silva, AJ et al*.*, 2021) [16]. The target PCR products were purified and sequenced with Sanger sequencing (First BASE Laboratories, Selangor, Malaysia).

### Full genome sequencing

Library preparation and target enrichment for full genome sequencing was performed using an Illumina RNA Prep with Enrichment with viral surveillance panel (Illumina, San Diego, CA, USA). Briefly, 8.5 µl of total nucleic acid was used for library preparation as described by the manufacturer’s protocol. First, cDNA was synthesized in two steps for first- and second-strand cDNA. Then, double-stranded cDNA was tagmented by using bead-linked transposomes (EBLTL) and purified. The tagmented fragments were amplified to add index by Illumina DNA/RNA UD Indexes. After clean-up, libraries were quantified using Invitrogen Qubit dsDNA BR assay kit (Thermo Fisher Scientific). The second step for library prep included using one-plex reactions for probe hybridization and oligos from the Illumina viral surveillance panel. Hybridized probes were then captured, washed, and amplified. Library quantity was determined with the Qubit 4 Fluorometer (Thermo Fisher Scientific) and QIAxcel Advanced System (QIAGEN, Hilden, Germany) (approximately 400–500 bps). Sequencing was performed on the Illumina MiSeq sequencer, using the MiSeq Reagent Kit v3 at 2 × 151 bps read length.

### Bioinformatic analysis

The partial RNA polymerase gene sequences of the PCR products were obtained from assembling forward and reverse reads of Sanger sequencing and trimming the primer regions in MEGA11 [17]. Subsequently, genotyping was performed using the Norovirus Typing Tool Version 2.0 [18] and Human Calicivirus Typing tool [19] The genotype was compared to the reference strains available in the GenBank database using the Basic Local Alignment Software Tool (BLAST) [20]. On the other hand, reads generated by full genome sequencing were filtered for high-quality reads by Trimmomatic v0.39 [21]. Taxonomic labels were assigned to the filtered reads by Kraken [22] and visualized with Krona [23]. The consensus genome was constructed using reference mapping by BWA v0.7.17-r1188 [24] and de novo assembly by SPAdes v3.12.0 [25]. High-quality reads were mapped to the top BLAST hit genome using BWA, and then the generated Sequence Alignment Map (SAM) file was converted into a draft genome by SAMtools [26]. The consensus genome was then obtained by aligning the scaffolds assembled by SPAdes to the draft genome.

### Phylogenetic analysis

Phylogenetic trees were constructed from nucleotide sequence alignments of the full genome sequence, partial ORF1 (RdRp region) and ORF2 genes (VP1 region) (corresponding to the positions 4840–5101 and 5085–5366 in the NCBI Reference Sequence: NC_029646.1, respectively) using the maximum likelihood method in IQ-TREE 2 software [27]. The best codon substitution models were determined by ModelFinder [28] and branch supports were approximated from 1000 replicates of Ultrafast Bootstrap [29]. The phylogenetic trees were visualized with MEGA11.

### Statistical analysis

A crude rate ratio was calculated by a diarrheal weekly incidence per 100,000 population during the outbreak which was divided by a five-year median of diarrheal weekly incidence per 100,000 population over the sporadic time. The Exact Poisson Method was used for calculating the 95%CI of the rate ratio, and the exact mid-P double-sided p-value was used for calculating a p-value. STATA version 16 was used for univariate analysis. Risk ratio, 95% confidence interval and p-value were used to assess whether food items were related to diarrheal incidence by using Chi-square test or Fisher exact tests based on expected values.

## Results

### Demographic data

From 26 December 2021 to 21 January 2022, the outbreak was first detected on 27 December 2022. The weekly rate of diarrheal cases on the first week of the outbreak was almost three times higher than the 5-year median weekly rates [RR (95%CI), 2.7 (2.0–3.7), *p*-value < 0.0001]. After that first week, the weekly rates fell below the 5-year median weekly rates. Specifically, the last two weekly rates were significantly lower than the 5-year median weekly rates [RR (95%CI), 0.3 (0.2–0.5), *p* < 0.0001]. Cases were reported from all 10 districts of Chanthaburi province and 40% of the patients lived within the Muang district.

Regarding the outbreak in the educational workshop, 52% of the medical students (19/36) reported diarrheal symptoms. Characteristics of these medical student patients were male to female ratio of 1 per 1.1, and a median age of 23 years. Among the received samples, there were 10 medical students and 4 healthcare workers, of which 35% (5/14) were positive for norovirus GII and none were positive for COVID-19. The median duration of sickness was 1 day (IQR 1–2 days), and the median duration from onset to collection date was 13 days (10–19 days). The latest norovirus case had symptom onset on 28 December 2021 and no case required hospitalization. There were four probable source menus (lunch, afternoon break, dinner, and night break before 23 December 2021); however, all food items were not statistically associated with observed cases. No food handlers reported symptoms of diarrhea and we did not test for norovirus among asymptomatic food handlers. Food preparation began 4 h before serving and food was kept at room temperature before serving.

### Norovirus detection in patients

Among the 93 samples tested, 42 (45%) samples were positive for diarrhea viruses tested by multiplex real-time RT-PCR (Allplex GI-Virus Assay), including 40 of 42 (95%) positive for norovirus GII with an average Ct value of 27.31 (min 12.92–max 39.96), with an additional one rotavirus and one norovirus and rotavirus co-infection. Of the 65 samples collected during the outbreak, 38 (58.5%) tested positive for norovirus GII with one multiple infection (norovirus GI, GII, and rotavirus), and one rotavirus-only (Fig. 1). The overall positive rate from the sporadic cases was 10% (3/28) (χ2 = 19.19, *p* < 0.001), where 3 positive samples were norovirus GII-positive including one GII.3[P16] genotype and two GII.4[P31] genotypes (Table 2).

**Table 2:** Norovirus GII classification in RdRp region and capsid region with the percentage of sequence identity from this study

Norovirus	Collection date	% Identity*	RdRp region	Capsid region	Accession no
PBH21022	29-Dec-21	98.9	GII.P25	GII.3	OQ300332
PBH21024	29-Dec-21	98.9	GII.P25	GII.3	OQ300333
PBH21028	26-Dec-21	98.3	GII.P25	GII.3	OQ300334
PBH22030	3-Jan-22	98.5	GII.P25	GII.3	OP954345
PBH22031	1-Jan-22	98.9	GII.P25	GII.3	OP954346
PBH22032	1-Jan-22	98.5	GII.P25	GII.3	OP954347
PBH22033	1-Jan-22	98.5	GII.P25	GII.3	OP954348
PBH22034	2-Jan-22	98.5	GII.P25	GII.3	OP954349
PBH22037	31-Dec-21	98.9	GII.P25	GII.3	OP954350
PBH22040	1-Jan-22	98.9	GII.P25	GII.3	OP954351
PBH22042	31-Dec-21	98.5	GII.P25	GII.3	OP954352
PBH22044	31-Dec-21	98.9	GII.P25	GII.3	OP954353
PBH22051	5-Jan-22	98.5	GII.P25	GII.3	OP954354
PBH22052	5-Jan-22	98.5	GII.P25	GII.3	OP954355
PBH22054	5-Jan-22	98.5	GII.P25	GII.3	OP954356
PBH22055	4-Jan-22	98.5	GII.P25	GII.3	OP954357
PBH22056	3-Jan-22	98.5	GII.P25	GII.3	OP954358
PBH22066	6-Jan-22	98.5	GII.P25	GII.3	OP954359
PBH22068	2-Jan-22	98.5	GII.P25	GII.3	OP954360
PBH22070	2-Jan-22	98.3	GII.P21(GII.Pb)	GII.21	OP954361
PBH22071	2-Jan-22	99.1	GII.P17	GII.17	OP954362
PBH22072	2-Jan-22	98.5	GII.P25	GII.3	OP954363
PBH22073	2-Jan-22	98.9	GII.P25	GII.3	OP954364
PBH22075	7-Jan-22	98.5	GII.P25	GII.3	OP954365
PBH22094	7-Jan-22	99.1	GII.P17	GII.17	OP954366
PBH22095	7-Jan-22	98.9	GII.P12**	GII.3	OP954367
PBH22096	11-Jan-22	98.9	GII.P25	GII.3	OP954368
PBH22108	13-Jan-22	98.5	GII.P25	GII.3	OP954369
PBH22139	21-Feb-22	98.7	GII.P16	GII.3	OP954370
PBH22211	27-Jul-22	98.5	GII.P31**	GII.4	OQ300335
PBH22247	12-Sep-22	97.9	GII.P31**	GII.4	OQ300336

*The identity was compared with public sequence of each genotype: OL451532 (GII.3[P25]), MK396776 (GII.21[P21]), MT344182 (GII.17[P17]), LC621120 (GII.3[P12]), LC597117 (GII.3[P16]), MN294766 (GII.4[P31])

**Norovirus typing was classified using the Human Calicivirus Typing tool [19]. These three samples were unassignable by the Norovirus Typing Toll Version 2 [18]

### Norovirus genotype and phylogenetic tree analysis

Norovirus GII positive samples (n = 41) were further characterized for their partial RNA polymerase region and capsid region genotypes by Sanger sequencing. Nucleotide sequencing was successful in 31 samples. From the 28 samples collected within the December 2021–January 2022 outbreak, norovirus GII.3 was the most common genotype in the capsid region (89.3%, 25/28) within this population, followed by 2 of GII.17 (7.1%) and 1 of GII.21 (3.6%) (Table 2). In the RNA polymerase gene region, GII.P25 (24/28, 85.7%) was the most common genotype followed by 2 of GII.P17 (7.1%), one each of GII.P21 (GII.Pb) and GII.P12 (Table 2). Moreover, we found one GII.3 [P16] and two samples of GII.4[P31] during the non-outbreak cases from 21 February 2022 to 12 September 2022, respectively (Table 2).

**Fig. 2:**
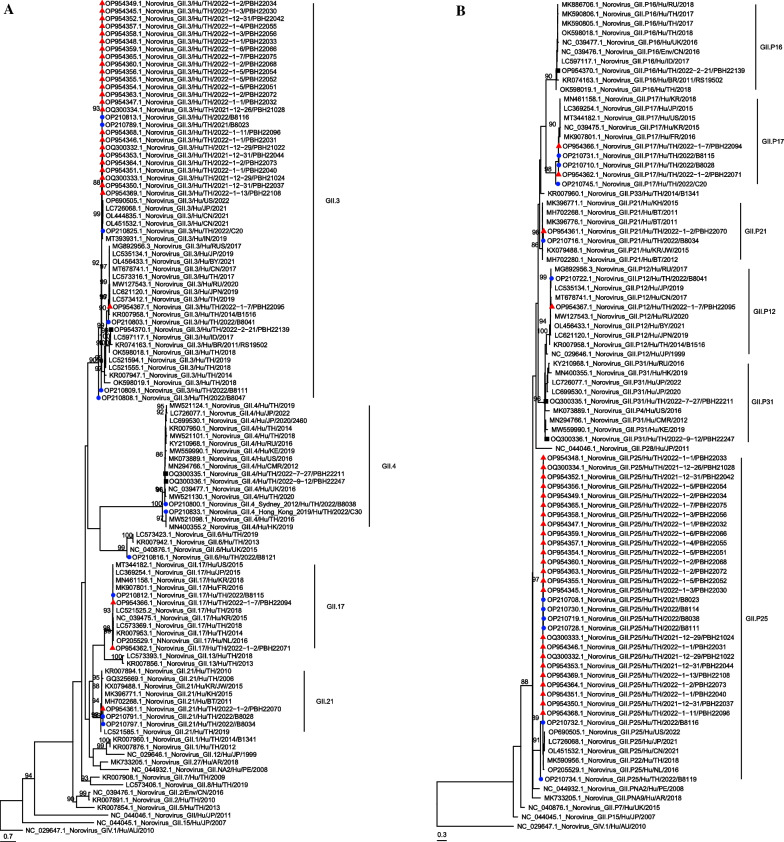
Phylogenetic analyses of the partial RdRp and VP1 regions of norovirus sequences. Phylogenetic analyses based on (**A**) the partial VP1 region (282 bp) and (**B**) the partial RdRp region (261 bp) of norovirus sequences isolated from patients in Chanthaburi province, Thailand from December 2021-September 2022. Red triangles represent sequences of 28 samples in the outbreak from December 2021 to January 2022 while black squares represent the other 3 sequences collected in 2022. Blue circles represent sequences from the previous study [12]. This tree was constructed with IQ-TREE2 using 1000 replicates of Ultrafast Bootstrap (shown only values greater than 85) and the best codon substitution model identified by ModelFinder (KOSI07 + FU + R3 and KOSI07 + FU + I + G4, respectively)

The phylogenetic tree of the partial RNA polymerase gene region (261 bp) and capsid region (282 bp) of detectable norovirus GII was constructed to investigate the relationship between the norovirus strains identified in this study and previous reports worldwide (Fig. 2). Detected norovirus showed nucleotide identity ranging from 97.9% to 99.1% compared to the public sequences of the same genotype, namely, OL451532 (GII.3[P25]), MK396776 (GII.21[P21]), MT344182 (GII.17[P17]), LC621120 (GII.3[P12]), LC597117 (GII.3[P16]) and MN294766 (GII.4[P31]). Phylogenetic analysis of VP1 region showed that GII.3 strains in the present study were in a distinct cluster from the global strain and Thailand strain isolated in 2014–2019 but clustered with the viruses found in China in 2021, USA 2022, Japan 2021, India 2019 and Thailand 2021–2022 (Fig. 2A). Phylogenetic analysis of the RdRp gene revealed that GII.P25 was grouped with norovirus isolated from Japan in 2021 (LC726068.1), China in 2021 (OL451532), USA in 2022 (OP690505), the Netherlands in 2016 (OP205529) and Thailand in 2018 (MK590956.1), We further demonstrate the presence of multiple co-circulating norovirus genotypes in Chanthaburi province, including GII.17[P17], GII.21[P21], GII.3[P12] and GII.4[P31]. The sequences from our study were deposited into the GenBank (National Center for Biotechnology Information, NCBI) with accession numbers OP954332–OP954370 and OQ300335–OQ300336.

### Whole genome sequencing

**Fig. 3:**
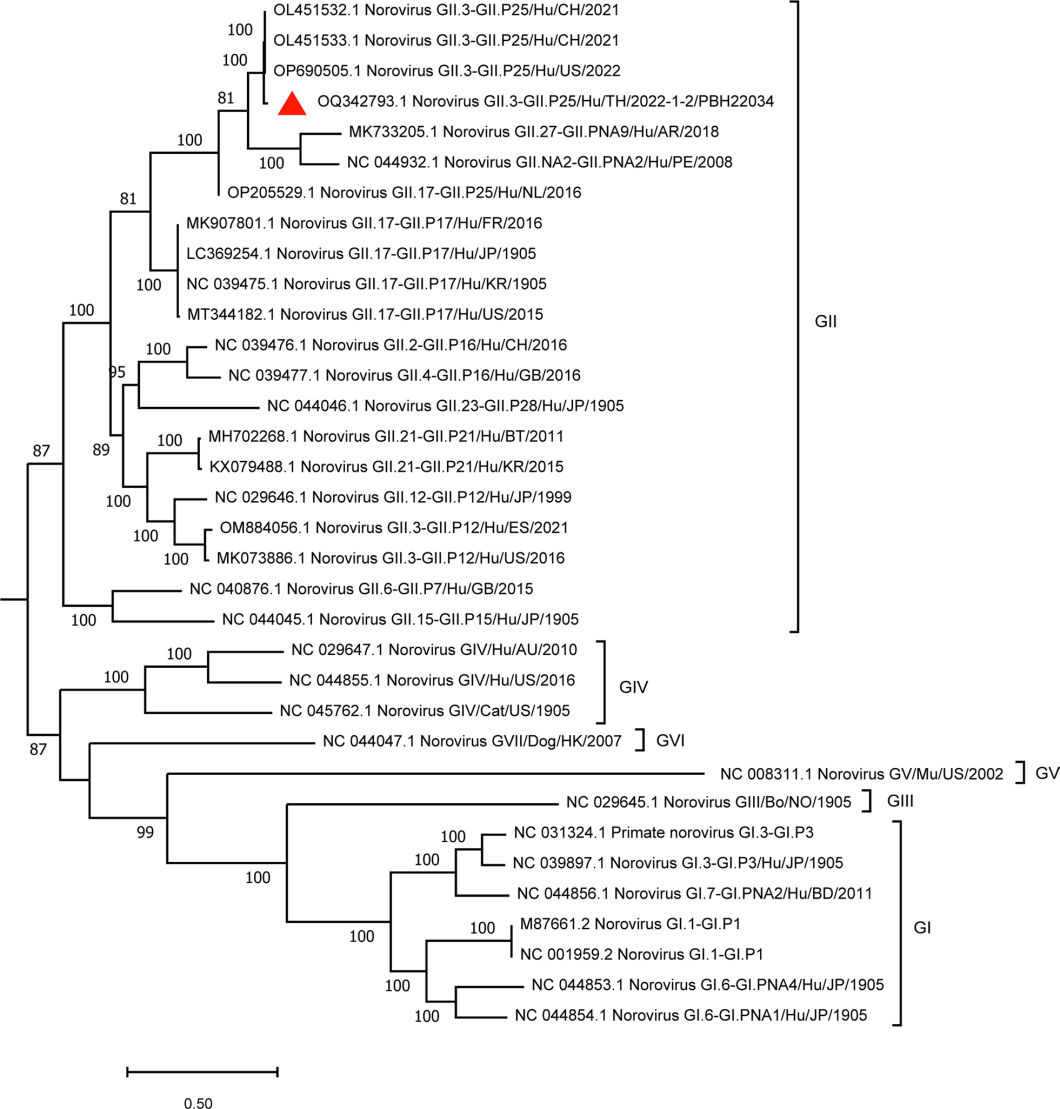
Phylogenetic analysis of the full genome sequence of norovirus obtained in this study. Red triangles represent sequences from this study (PBH22034). This tree was constructed with IQ-TREE2 using 1000 replicates of Ultrafast Bootstrap (shown only values greater than 80) and the best codon substitution model identified by ModelFinder (UNREST + FO + I + G4)

From the norovirus genotype analysis, GII.3P[25] was selected for whole-genome sequencing in this study because (1) it was the dominant norovirus strain during the outbreak; (2) it was the first time this strain was reported in Thailand; and (3) it demonstrated atypical epidemiology by primarily infecting adults (21 patients were > ten years old, 3 were less than 10 years old). Full genome sequencing of a norovirus GII.3[P25] positive specimen (PBH22034) generated a total of 2,187,506 high-quality reads which included 220,341 norovirus reads assigned by Kraken. The full genome sequence was assembled by using OL451533.1 as a reference sequence resulting in a full genome (7584 nucleotide) with 28,559 × average coverage depth (with at least 300 × read supports per base). The scaffold assembled by de novo assembly also matches with the genome at position 68–7584. This genome sequence contained three open reading frames: ORF1 (5,136 nt), ORF2 (1,647 nt), and ORF3 (765 nt). Using the same genotyping tools as above, this sequence was characterized as a GII.3[P25] strain. The full genome sequence was deposited into the NCBI GenBank with an accession number of OQ342793. Phylogenetic analysis of the full genome sequence was constructed with IQ-TREE2 using the best nucleotide substitution model identified by ModelFinder (UNREST + FO + I + G4). The tree is shown in Fig. 3. It showed 98.62% and 98.57% similarity to the norovirus from China detected in 2021 and USA detected in 2022, respectively.

## Discussion

This study sought to investigate the genetic diversity of norovirus during a short outbreak period, December 2021 to January 2022, and subsequent sporadic cases in eastern Thailand through September 2022. Unlike previous norovirus outbreaks, which typically affect young children under the age of 5, this outbreak included a cluster of medical students who ranged in age 21 to 23. Infants and children are naive to norovirus exposure at birth and common behaviors, including the repetitive touching of surfaces or objects in the mouth and poor hygiene, often leads to norovirus infection [30]. More than 50 specimens in this study were collected from infants and children less than 10 years; 21 specimens (42%) were positive for norovirus GII. However, the main cluster from this study who tested positive for norovirus GII was adults > 20 years old; 19 of 37 (51.35%) were positive. Norovirus GII was the predominant pathogen of this outbreak.

A diarrheal disease outbreak was identified in a workshop of 36 medical students during the winter months. The exposure time was suspected to be on December 23, 2022. Recall bias might affect the subsequent outbreak investigation; however, we used menu lists from each day of the workshop to lessen the impact of bias. The probable source of the outbreak was fresh vegetables served in the lunch box; however, there was no food remaining and specimens were not collected from the chefs, assistants, and staff. Several diarrheal cases in Chanthaburi Province had an exposure history to large fresh markets in the Muang district, and the suspected source was food, including fresh vegetables, berries, and fruits. The environmental investigations for the source of infection from the other study found 8/24 produce samples (such as salad greens, cabbage, cucumber, and tomato) and ice were norovirus-positive, and GII.3[P25] was identified in a tomato [12].

Among the Norovirus GII-positive samples, 31 were successfully dual-typing sequenced in the polymerase (RdRp region) and capsid (VP1 region) genes using two genotyping tools, including the using the Norovirus Typing Tool Version 2.0 [18] and the Human Calicivirus Typing tool [19]. Interestingly, three samples (PBH22095, − 22,211 and − 22,247) were characterized as unassignable when using Norovirus Typing Tool Version 2.0 at the RdRp region but they were characterized as GII.P12 and two of GII.P31, respectively (Table 2) and they were confirmed with the phylogenetic tree analysis (Fig. 2B). Multiple genotyping tools are essential if the unassignable or novel strain is detected. The RdRp and VP1 regions of the norovirus genome have the most variation and recombination [15]. Targeted sequencing within these regions is currently the best strategy for variant characterization. Moreover, the construction of phylogenetic trees during and after the outbreak (Fig. 2) shows the evolution of norovirus variants during outbreaks and strain emergence via recombination and can provide insights into future outbreaks. During the outbreak, norovirus GII.3 (89.3%) was the most common capsid region genotype, and GII.P25 (85.7%) was the predominant polymerase region genotype, with GII.3[P25] is the predominant strain (85.7%). Additionally, recombination between strains and point mutations resulted in changes in genetic diversity, and recombinant variants might be more infectious and virulent than the prototype strains [7, 31]. However, a severe case of the GII.3[P25] strain was not reported in our study. This may be explained due to most patients being adults and symptoms were expectedly mild.

From 2000 to 2019, norovirus GII.4 was the most prevalent genotype circulating in Thailand [9, 10], while GII.3[P25] was first reported in 2021 from patients and produced concurrent with our study [12]. However, fourteen genotypes were detected among 30 norovirus GII positive cases, including GII.3[P25] (9/30), GII.6[P7] (3/30); 2 each of GII.3[P7], GII.3[P12], GII.17[P17], GII.21[P17], and GII.21[P21]; and 1 each of GII.3[P17], GII.3[P31], GII.4 Sydney[P7], GII.4 Sydney[P25], GII.4 Hong Kong [P7], GII.4 Hong Kong [P31], GII.6[P17], and GII.7[P7]. Genotype GII.3[P25] accounted for one-third (30%) of cases from a previous study [12] even though within our study 85.7% of tested specimens were GII.3[P25], and it was the overwhelmingly predominant strain responsible for the outbreak cluster (Χ^2^ = 5.13, *p* < 0.05). Four norovirus genotypes were identified during the Dec 2021–Jan 2022 outbreak from our study, including GII.3[P25] (24/28, 85.7%), GII.17[P17] (2/28, 7.1%), and each of GII.21 [P21], and GII.3[P12]. Interestingly, the RdRp region of our GII.3[P25] strains show 98.7% identity (231 bp) with GII.4 Sydney[P25] sequence (OP210719.1, sample collected on 7 Jan 2022) from the previous study [12]. With high sequence identity, GII.4 Sydney[P25] is possibly a recombinant of GII.3[P25] with GII.4 strain. Further studies on recombination analysis using the full sequences to identify the recombination breakpoints and determine the recombination characteristic are essential to elucidate this observation. Sampling size and location might have accounted for the different findings, but both studies showed GII.3[P25] as a significant cause of the outbreak. In addition, Chuchaona et al*.* [12] mentioned low viral loads (Ct ≥ 30) for many of the samples included in their study, while high viral loads were found in our study with an average Ct value of 27.31 (min 12.92–max 39.96).

Three other norovirus strains (GII.17[P17], GII.21[P21], and GII.3[P12]) co-circulating during the outbreak were commonly found in Thailand [9–12]. Interestingly, after the outbreak, the three positive viral diarrhea cases were infected with previous circulating norovirus strains; GII.3[P16] and GII.4[P31] (Table 2). Until September 2022 (the last month of the study), infection with GII.3[P25] was not detected in this hospital and was no longer circulating in the community. The GII.3[P25] strain is responsible for only one outbreak and is not widely circulating in Thailand. Nonetheless, it is important to continuously monitor the molecular epidemiology of noroviruses in other regions of Thailand. Norovirus GII.4 has been the predominant norovirus strain circulating in Thailand and worldwide. In this study, we found norovirus GII.4[P31] in only two samples outside the outbreak period (Table 2). Norovirus GII.4[P31] strain was reported in Thailand in 2018 [32] and caused an outbreak in Japan in 2021[33]. Norovirus GII.3[P16] strain was found in one sample in February 2022; it was first detected in Thailand in 2018 [34]. These results highlight the genetic diversity of circulating norovirus GII genotypes in Thailand during the outbreak and sporadic cases and emphasize the importance of continuous molecular surveillance of circulating noroviruses in the community.

Full genome sequencing is a powerful tool for the detection, identification, and discrimination of norovirus strains. In our study, we selected one unique strain of norovirus GII.3[P25] from the outbreak for conducting full genome sequencing. The full genome and Sanger sequences indicated the same genotype (Fig. 3). Full genome sequence of norovirus GII.3[P25] from our study is the first reported in Thailand. The first GII.3[P25] was reported from India in January 2019 as a partial sequence in GenBank (accession no. MT393931.1, unpublished journal). There are three full genome sequencesof norovirus GII.3[P25] available in NCBI GenBank (accessed on 22 October 2023). The first two complete genomes were reported from China in March 2021, (accession no. OL451532.1 and OL451533.1, unpublished journal) and recently from the USA in March 2022 (accession no. OP690505, unpublished journal). Our additional data could provide insights into the viral evolution, allowing for more accurate predictions and appropriate response measures in future outbreaks.

The COVID-19 pandemic demonstrated that using NPIs such as global lockdowns, social distancing, awareness of hygiene, handwashing, disinfection, and the wearing of face masks can also reduce norovirus transmission [4]. However, the use of alcohol-based hand sanitizers, having limited efficacy against noroviruses, is not recommended as a preventative measure for viral gastroenteritis. Alcohol cannot eliminate the norovirus due to its lack of a viral envelope. Handwashing is a suitable measure for eliminating norovirus [35]. This outbreak occurred during New Years Eve and the relaxation of COVID-19 restrictions in Thailand when people often celebrate with co-workers, family, and friends. A recent report from the same outbreak detected norovirus GII.3[P25] from tomatoes at Chanthaburi during the same period of the outbreak but there was no traceable link between patients and produce [12]. To prevent the spread of norovirus, we suggest cleaning vegetables with running water and hand hygiene prior to eating or cooking [36].

As a result of this study, we recommend continued environmental investigations into the source of norovirus exposure during outbreaks, including sampling people involved in the processing of food (i.e. ice factory workers, chefs, and restaurant staff). Furthermore, we emphasize the importance of early detection of abnormal numbers of diarrheal cluster via event-based surveillance and maintaining the quality of water for hand and food cleaning and for use in ice production. It is important to communicate the risk about diarrheal disease during cool season, and promote norovirus prevention strategies, including hand hygiene and proper cleaning of vegetables with running water prior to eating or cooking food at least 90 °C for 90 s [37] to prevent future outbreaks.

## Conclusions

Several pieces of evidence show this unusual norovirus outbreak, including (1) a higher infection rate than the median of the past five years and 60% and 10% positive rates during and after the outbreak, respectively; (2) adults were the primary infected population rather than children; (3) GII.3[P25] was first detected in Thailand and (4) the predominant cause of diarrhea outbreak in this cluster. GII.3[P25], the dominant genotype from our study, could suggest the genotype's ability to lead to independent outbreaks, although more investigation is necessary. The first full genome sequence of GII.3[P25] from Thailand was obtained and clustered within the same lineage from China with 98.62% nucleotide similarity. A suggested source of the outbreak was contaminated vegetables. Moreover, routine surveillance of circulating noroviruses in the community continues to be essential for detecting, preventing, and controlling future viral diarrheal disease outbreaks and the need for further support of ongoing vaccine development programs.

## Data Availability

All data generated or analyzed during this study are included in this published article.

## References

[1] Nguyen GT, Phan K, Teng I, Pu J, Watanabe T (2017). A systematic review and meta-analysis of the prevalence of norovirus in cases of gastroenteritis in developing countries. Medicine (Baltimore).

[2] Deval J, Jin Z, Chuang Y-C, Kao CC (2017). Structure(s), function(s), and inhibition of the RNA-dependent RNA polymerase of noroviruses. Virus Res.

[3] Campillay-Véliz CP, Carvajal JJ, Avellaneda AM, Escobar D, Covián C, Kalergis AM (2020). Human norovirus proteins: implications in the replicative cycle, pathogenesis, and the host immune response. Front Immunol.

[4] O’Reilly KM, Sandman F, Allen D, Jarvis CI, Gimma A, Douglas A (2021). Predicted norovirus resurgence in 2021–2022 due to the relaxation of nonpharmaceutical interventions associated with COVID-19 restrictions in England: a mathematical modeling study. BMC Med.

[5] Alsved M, Fraenkel C-J, Bohgard M, Widell A, Söderlund-Strand A, Lanbeck P (2019). Sources of airborne norovirus in hospital outbreaks. Clin Infect Dis.

[6] Parra GI (2019). Emergence of norovirus strains: a tale of two genes. Virus Evolut..

[7] Chhabra P, de Graaf M, Parra GI, Chan MC-W, Green K, Martella V (2019). Updated classification of norovirus genogroups and genotypes. J General Virol.

[8] Cuevas JM, Combe M, Torres-Puente M, Garijo R, Guix S, Buesa J (2016). Human norovirus hyper-mutation revealed by ultra-deep sequencing. Infect Genet Evol.

[9] Kumthip K, Khamrin P, Maneekarn N (2018). Molecular epidemiology and genotype distributions of noroviruses and sapoviruses in Thailand 2000–2016: a review. J Med Virol.

[10] Thanusuwannasak T, Puenpa J, Chuchaona W, Vongpunsawad S, Poovorawan Y (2018). Emergence of multiple norovirus strains in Thailand, 2015–2017. Infect Genet Evol.

[11] Boonyos P, Boonchan M, Phattanawiboon B, Nonthabenjawan N, Tacharoenmuang R, Gunpapong R (2021). Spread of genetically similar noroviruses in Bangkok, Thailand, through symptomatic and asymptomatic individuals. Heliyon.

[12] Chuchaona W, Khongwichit S, Luang-On W, Vongpunsawad S, Poovorawan Y (2023). Norovirus GII. 3 [P25] in patients and produce, Chanthaburi Province, Thailand 2022. Emerg Infect Dis.

[13] Douglas A, Sandmann FG, Allen DJ, Celma CC, Beard S, Larkin L (2021). Impact of COVID-19 on national surveillance of norovirus in England and potential risk of increased disease activity in 2021. J Hosp Infect.

[14] Nachamkin I, Richard-Greenblatt M, Yu M, Bui H (2021). Reduction in sporadic norovirus infections following the start of the COVID-19 pandemic, 2019–2020. Philadelphia Infect Dis Ther.

[15] Chhabra P, Browne H, Huynh T, Diez-Valcarce M, Barclay L, Kosek MN (2021). Single-step RT-PCR assay for dual genotyping of GI and GII norovirus strains. J Clin Virol.

[16] Silva AJ, Yang Z, Wolfe J, Hirneisen KA, Ruelle SB, Torres A (2021). Application of whole-genome sequencing for norovirus outbreak tracking and surveillance efforts in Orange County. CA Food Microbiol.

[17] Tamura K, Stecher G, Kumar S (2021). MEGA11: molecular evolutionary genetics analysis version 11. Mol Biol Evol.

[18] Kroneman A (2011). An automated genotyping tool for enteroviruses and noroviruses. J Clin Virol.

[19] Tatusov RL, Chhabra P, Diez-Valcarce M, Barclay L, Cannon JL, Vinjé J (2021). Human Calicivirus Typing tool: a web-based tool for genotyping human norovirus and sapovirus sequences. J Clin Virol.

[20] Altschul SF, Gish W, Miller W, Myers EW, Lipman DJ (1990). Basic local alignment search tool. J Mol Biol.

[21] Bolger AM, Lohse M, Usadel B (2014). Trimmomatic: a flexible trimmer for Illumina sequence data. Bioinformatics.

[22] Wood DE, Salzberg SL (2014). Kraken: ultrafast metagenomic sequence classification using exact alignments. Genome Biol.

[23] Ondov BD, Bergman NH, Phillippy AM (2011). Interactive metagenomic visualization in a Web browser. BMC Bioinform.

[24] Li H, Durbin R (2009). Fast and accurate short read alignment with Burrows–Wheeler transform. Bioinformatics.

[25] Bankevich A, Nurk S, Antipov D, Gurevich AA, Dvorkin M, Kulikov AS (2012). SPAdes: a new genome assembly algorithm and its applications to single-cell sequencing. J Comput Biol.

[26] Danecek P, Bonfield JK, Liddle J, Marshall J, Ohan V, Pollard MO (2021). Twelve years of SAMtools and BCFtools. Gigascience..

[27] Minh BQ, Schmidt HA, Chernomor O, Schrempf D, Woodhams MD, von Haeseler A (2020). IQ-TREE 2: new models and efficient methods for phylogenetic inference in the genomic era. Mol Biol Evol.

[28] Kalyaanamoorthy S, Minh BQ, Wong TKF, von Haeseler A, Jermiin LS (2017). ModelFinder: fast model selection for accurate phylogenetic estimates. Nat Methods.

[29] Hoang DT, Chernomor O, von Haeseler A, Minh BQ, Vinh LS (2017). UFBoot2: improving the ultrafast bootstrap approximation. Mol Biol Evol.

[30] Cannon JL, Lopman BA, Payne DC, Vinjé J (2021). Corrigendum to: birth cohort studies assessing norovirus infection and immunity in young children: a review. Clin Infect Dis.

[31] Pabbaraju K, Wong AA, Tipples GA, Pang XL (2019). Emergence of a novel recombinant norovirus GII.P16-GII.12 strain causing gastroenteritis, Alberta Canada. Emerg Infect Dis.

[32] Khamrin P, Kumthip K, Yodmeeklin A, Jampanil N, Phengma P, Yamsakul P (2022). Changing predominance of norovirus recombinant strains GII.2[P16] to GII.4[P16] and GII.4[P31] in Thailand, 2017 to 2018. Microbiol Spectr..

[33] Pham NTK, Nishimura S, Shimizu-Onda Y, Trinh QD, Komine-Aizawa S, Khamrin P (2022). Emerging norovirus GII.4 Sydney[P31] causing acute gastroenteritis outbreak in children in Japan, during COVID-19. J Infect Chemother.

[34] Khamrin P, Kumthip K, Yodmeeklin A, Jampanil N, Phengma P, Yamsakul P (2022). Changing predominance of norovirus recombinant strains GII. 2 [P16] to GII. 4 [P16] and GII. 4 [P31] in Thailand, 2017 to 2018. Microbiol Spectrum..

[35] Cannon JL, Bonifacio J, Bucardo F, Buesa J, Bruggink L, Chan MC (2021). Global trends in norovirus genotype distribution among children with acute gastroenteritis. Emerg Infect Dis.

[36] Hall AJ, Vinjé J, Lopman B, Park GW, Yen C, Gregoricus N (2011). Updated norovirus outbreak management and disease prevention guidelines. Morb Mortal Wkly Rep.

[37] Codex Alimentarius. CAC/GL 79-2012 guidelines on the application of general principles of food hygiene to the control of viruses in food. Codex Committee on Food Hygiene. 2012;13.

